# Exploring the relationship among dispositional optimism, health-related quality of life, and CIPN severity among colorectal cancer patients with chronic peripheral neuropathy

**DOI:** 10.1007/s00520-021-06352-0

**Published:** 2021-07-07

**Authors:** Hester.R. Trompetter, Cynthia S. Bonhof, Lonneke V. van de Poll-Franse, Gerard Vreugdenhil, Floortje Mols

**Affiliations:** 1 CoRPS - Center of Research on Psychological and Somatic Disorders, Department of Medical and Clinical Psychology, Tilburg University, P.O. Box 90153, 5000 LE Tilburg, The Netherlands; 2 Department of Research, Netherlands Comprehensive Cancer Organisation (IKNL), Utrecht, The Netherlands; 3 Division of Psychosocial Research and Epidemiology, The Netherlands Cancer Institute, Amsterdam, The Netherlands; 4 Department of Internal Medicine, Máxima Medical Centre, Eindhoven and Veldhoven, The Netherlands

**Keywords:** Cancer, Cancer pain, CIPN, Health-related quality of life, Optimism, Peripheral neuropathy

## Abstract

**Purpose:**

Chemotherapy-induced peripheral neuropathy ((CI)PN) becomes chronic in 30% of cancer patients. Knowledge of predictors of chronic (CI)PN and related impairments in health-related quality of life (HRQoL) is lacking. We examined the role of optimism in chronic (CI)PN severity and associated HRQoL in colorectal cancer (CRC) patients up to two years after diagnosis.

**Methods:**

CRC patients from a prospective cohort study participated, with sensory peripheral neuropathy (SPN) 1 year after diagnosis (*n* = 142). Multivariable regression analyses examined the cross-sectional association between optimism (measured by the LOT-R) and SPN severity/HRQoL (measured by the EORTC QLQ-CIPN20 and QLQ-C30), as well as the prospective association in a subsample that completed measures 2 years after diagnosis and still experienced SPN (*n* = 86).

**Results:**

At 1-year follow-up, higher optimism was associated with better global HRQoL, and better physical, role, emotional, cognitive, and social functioning (all *p* < .01). Optimism at year one was also prospectively associated with better global HRQoL (*p *< .05), and emotional and cognitive functioning at 2-year follow-up (both *p* < .01). Optimism was not related to self-reported SPN severity. Significant associations were retained when controlling for demographic/clinical variables, and became non-significant after controlling for depressive and anxiety symptoms.

**Conclusions:**

Optimism and depressive and anxiety symptoms are associated with HRQoL in CRC patients with chronic (CI)PN. Future research may illuminate the mechanisms that these factors share, like the use of (non)adaptive coping styles such as avoidance and acceptance that may inform the design of targeted interventions to help patients to adapt to chronic (CI)PN.

## Introduction

Chemotherapy-induced peripheral neuropathy ((CI)PN) often presents as sensory, motor, and/or autonomic symptoms in the hands and feet that include tingling, numbness, and neuropathic pain [1]. In 30% of cancer patients, (CI)PN becomes a *chronic condition* that persists for 6 months or longer after the cessation of chemotherapy [2]. In colorectal cancer (CRC), (CI)PN often results from the administration of oxaliplatin during chemotherapy and is primarily sensory [3, 4]. CRC patients report (CI)PN up to 11 years after the cessation of chemotherapy [3, 4], and may experience severe long-term impairments in health-related quality of life (HRQoL) as a result of (CI)PN [3, 5, 6].

Unfortunately, it is difficult to effectively treat (CI)PN [7]. Knowledge of potentially modifiable predictors of chronic (CI)PN and related impairments in HRQoL may help to find (non)pharmacological treatment opportunities. Predictors of acute (CI)PN include type and cumulative dosage of chemotherapy agent received, pretreatment neuropathy, age, and comorbid conditions like diabetes or rheumatoid arthritis [8, 9]. Not many studies focus on predictors of chronic (CI)PN and associated impairments in HRQoL [10, 11]. In parallel to other chronic pain-related syndromes, it is likely that *psychosocial predictors* play an important role [12, 13]. Biopsychosocial models of chronic pain agree that, over time, patient’s symptom interpretation will influence coping styles and behavior patterns like repetitious avoidance of activities that may increase symptoms, and subsequently, long-term HRQoL. After the transition to chronic (CI)PN, psychosocial predictors may thus gain in importance in comparison to the known—often biomedical and clinical—predictors of (acute) (CI)PN. A cross-sectional study of CRC patients with chronic (CI)PN that were assessed on average 5.6 years after diagnosis showed that anxiety and depressive symptoms exacerbated (CI)PN-related fatigue [10]. Other studies found evidence for the role of pre-treatment anxiety symptoms, perceived stress, and mindfulness in (CI)PN symptom intensity and components of HRQoL [11, 14, 15].

This study examines the role of *dispositional optimism* in chronic (CI)PN symptom severity and associated HRQoL in a sample of CRC patients. Dispositional optimism refers to a generalized tendency to expect future positive outcomes [16]. Systematic reviews summarize findings on the beneficial role of optimism in pain and adjustment to chronic illness [17, 18]. Optimists tend to face problems and maintain goal-directed efforts in challenging circumstances—such as chronic PN—due to their high perceived probability of success. As a consequence, they use more approach than avoidance coping styles and show lower levels of catastrophizing thinking [19]. Studies in cancer patients reported that optimism is cross-sectionally and prospectively associated with lower levels of (post-operative) pain and higher levels of HRQoL (27–29). To the best of our knowledge, however, no studies exist on the role of optimism in (CI)PN.

We use data from the longitudinal PROCORE study that assessed CRC patients before treatment and at one- and 2-year follow-up. A recent publication on the course of (CI)PN using this dataset revealed that 57% and 47% of the sample that was treated with chemotherapy experienced clinically relevant sensory peripheral neuropathy (SPN) one and 2 years after diagnosis, respectively [20]. SPN was prospectively associated with significantly worse global HRQoL and worse physical, role, emotional, cognitive, and social functioning over time. Concretely, we will investigate if dispositional optimism among CRC patients with SPN symptoms measured 1 year after diagnosis influences SPN symptom severity and HRQoL at one and 2 years after diagnosis. We hypothesize that dispositional optimism in this sample is cross-sectionally and prospectively associated with SPN symptom severity and HRQoL, also beyond other relevant demographic, clinical, and psychological factors including anxiety and depressive symptoms.

## Methods

### Setting, participants, and procedure

The PROCORE study is a prospective, population-based cohort study among CRC patients. PROCORE aims to examine the longitudinal impact of CRC and its treatment on patient-reported outcomes. Patients who were newly diagnosed with CRC as a primary tumor between January 2016 and January 2019 in four Dutch hospitals were invited to participate. Patients were excluded from participation when then they had (1) a previous cancer diagnosis (except for basal cell carcinoma), (2) cognitive limitations, and/or (3) an inability to read or write Dutch. Eligible patients were included shortly after diagnosis, before the start of initial treatment. The PROCORE study was approved by the certified Medical Ethic Committee of Medical research Ethics Committees United (registration number: NL51119.060.14).

Details of the data collection procedure have been described previously [20]. Data collection was performed via the PROFILES registry (www.profilesregistry.nl). PROFILES was set up to study the physical and psychosocial impact of cancer and its treatment [21]. Eligible participants were invited to participate in the study via their research nurse or case manager. They received an information package that contained an information letter and informed consent form, as well as the baseline questionnaire (wave 1). Participants received a follow-up questionnaire 4 weeks after surgery (wave 2; only when applicable), and 1 year (wave 3) and 2 years (wave 4) after diagnosis.

As we focused on *chronic* (CI)PN and investigated if dispositional optimism measured at 1-year follow-up influenced outcomes at one- and 2-year follow-up, we included patients who minimally completed the questionnaire 1 year after diagnosis. In practice, a number of patients were included in the study who had a previous cancer diagnosis and/or already started treatment. Parallel to previous studies based on the PROCORE dataset [20], patients were thus excluded from data analysis if they were previously diagnosed with cancer and reported baseline EORTC QLQ-CIPN20 scores > 0 and/or had already started chemotherapy at time of baseline. Finally, we solely focused on SPN and not motor/autonomic peripheral neuropathy symptoms, as SPN is most prevalent in CRC and distinguishes between CRC patients with peripheral neuropathy who are (not) treated with chemotherapy [3, 20, 22, 23]. Therefore, patients were included if they experienced some level of SPN at 1-year follow-up (EORTC QLQ-CIPN20 SPN subscale score > 0, [24]). Patients with CIPN20 SPN scores > 0 were not included if they only experienced hearing problems as it has been found unlikely that the item can accurately identify peripheral neuropathy [25].

### Measurements

#### Sociodemographic and clinical characteristics

Sociodemographic characteristics included age, sex, educational level, and partnership status. Clinical characteristics included tumor location, clinical stage, tumor differentiation grade, and treatment received. Comorbidity was assessed with the adapted Self-administered Comorbidity Questionnaire [26].

#### SPN

The EORTC QLQ-CIPN20 SPN subscale was used to assess SPN [24]. Six questions inquire to which extent during the past week someone experienced tingling, numbness, or shooting/burning pain in either the fingers and hands, or toes and feet. Three additional questions inquire the extent to which someone experienced problems standing or walking due to difficulty feeling feet, distinguishing between hot and cold, or experienced hearing problems. Answers are given on a 4-point Likert scale. Scores were linearly transformed to a 0–100 scale, with higher scores representing higher levels of SPN symptom severity.

#### Optimism

The Life Orientation Test-Revised (LOT-R) is a widely used measure of dispositional optimism [27]. The LOT-R consists of three positively formulated items (e.g., “In uncertain times, I usually expect the best”) and three negatively formulated items (e.g., “I hardly ever expect things to go my way”). Four additional filler items are not included in the final scale. Items are scored on a 5-point Likert scale. The negatively formulated items are reverse-coded before calculating a total scale score, where higher scores represent higher levels of optimism.

#### HRQoL

The EORTC QLQ-C30 was used to assess HRQoL [28]. In this study, we used the global health status/QoL scale and the five functioning scales (physical, emotional, social, role, and cognitive functioning). Questions about global health status are answered on a 7-point Likert scale; all other questions are answered on a 4-point Likert scale. Scales scores were linearly transformed to a 0–100 scale, with higher scores representing either better QoL or functioning.

#### Depressive and anxiety symptoms

The Hospital Anxiety and Depression Scale (HADS, 33) was used to control for potential psychological confounders in the study [10, 14]. The HADS assesses the presence and severity of anxiety (seven items) and depressive symptoms (seven items). All questions are answered on a 4-point Likert scale. Higher scores indicate higher anxiety or depressive symptoms (range for each of the subscales 0–21).

### Statistical analyses

The Statistical Program for Social Sciences (IBM SPSS) version 26.0 was used for all statistical analyses. Frequency distributions were calculated and descriptive analyses were performed to summarize sample characteristics. Chi-square tests and independent *t* tests allowed us to compare characteristics of our sample with the excluded patients that also completed the questionnaire at 1-year follow-up but did not experience SPN. We also calculated frequency distributions for the separate SPN symptoms to gain a better understanding of the nature and severity of SPN in the sample. These frequency distributions were calculated in the full sample as well as the subsample with patients who responded to, and still experienced SPN symptoms at, 2-year follow-up (wave 4; i.e., experienced some level of SPN as indicated by an EORTC QLQ-CIPN20 SPN subscale score > 0 that were not solely hearing problems). To examine the effect of optimism (independent variable) on SPN symptom severity, global HRQoL, and the five HRQoL functioning scales (dependent variables), multivariable stepwise regression analyses were performed. The first step of the regression analyses included LOT-R scores, followed by a second step in which control variables were added that were chosen based on previous findings on the course of PN and its association with HRQoL in the PROCORE dataset [20]. These variables included age, sex, education level (high vs. low/medium), partnership status, and disease stage (III and IV vs. other). In a third and final step, we included depression and anxiety scores as measured at 1-year follow-up to control for potential psychological confounders [10, 14]. Several outliers existed at 1-year follow-up on the QLQ-CIPN20 SPN subscale (*n* = 3) and the emotional functioning subscale of the QLQ-C30 (*n* = 6). As removal of these outliers from the regression analyses did not change the outcomes, the results of analyses including these outliers were reported.

Finally, we wanted to assess the prospective association of optimism with SPN symptom severity and HRQoL. We thus performed similar multivariable stepwise regression analyses as described above and examined if optimism measured at 1-year follow-up was associated with SPN symptom severity and HRQoL at 2-year follow-up beyond control variables (measured during 1-year follow-up). Subgroup data were used for these final analyses including the patients who responded to 2-year follow-up and still experienced SPN symptoms (excluding patients that solely reported hearing problems). One outlier existed at 2-year follow-up on the QLQ-CIPN20 SPN subscale. As removal of this outlier from the regression analyses did not change the outcomes, the results of analyses including this outlier were reported. An alpha level of 0.01 was applied to accommodate for multiple comparisons in both multivariable regression analyses. P values below 0.05 were deemed “marginally significant.”

## Results

### Patient characteristics

Of the 477 patients who completed the questionnaire at baseline, 77.6% (*n* = 370) completed the questionnaire at 1-year follow-up and 72.1% (*n* = 344) at 2-year follow-up. A full flow chart of the study has previously been published [20]. Of the 370 patients who completed the questionnaire at 1-year follow-up, 30 patients were excluded as they were previously diagnosed with cancer and reported baseline EORTC QLQ-((CI)PN)20 scores > 0 and/or had already started chemotherapy at time of baseline. Finally, 142 of the remaining 340 patients experienced some level of SPN symptoms other than solely hearing problems, and were included in the study.

Compared with the 198 patients out of the remaining 340 patients who also completed 1-year follow-up but did not experience SPN, patients in our sample received chemotherapy significantly more often (46.5 vs. 17%; *p* < 0.001). No other significant differences on demographic and clinical variables existed (data not shown). Patients in our sample were on average 67.0 years (SD = 8.9) and primarily male (61%), having a partner (84%), received medium education (60%), and living with one or more comorbid diseases (70%). Most patients suffered from a colon carcinoma (73%) in stage III (52%), and had undergone surgery (97%) (Table 1).

**Table 1:** Sociodemographic and clinical characteristics of CRC patients with PN, 1 year after diagnosis (*n* = 142)

	N (%)
Age (mean, SD)	67.1 (8.9)
Female sex	56 (39%)
Partner (yes)	118 (84%)
Education level^a^	
Low Medium High	16 (11%) 85 (60%) 41 (29%)
Tumor location	
Colon Rectum/rectum sigmoid Colon and rectum sigmoid	103 (73%) 36 (25%) 3 (2%)
TNM stage	
I II III IV Unknown	31 (22%) 29 (20%) 74 (52%) 5 (4%) 3 (2%)
Tumor differentiation grade	
Well differentiated Moderately differentiated Poorly differentiated Unknown	0 (0%) 114 (80%) 7 (5%) 21 (15%)
Radiotherapy (yes)	24 (17%)
Surgery (yes)	137 (97%)
Chemotherapy (yes)	66 (47%)
Number of comorbidities	
None One Two or more	42 (30%) 48 (34%) 50 (36%)
Most frequent comorbidities associated with PN	
Osteoarthritis Rheumatoid arthritis Diabetes mellitus	32 (23%) 8 (6%) 16 (11%)

Variables may deviate from 100% due to rounding off

abbreviations *CRC*, colorectal cancer; *(S)PN*, (sensory) peripheral neuropathy; *SD*, standard deviation

^a^Education level: low (no/primary school); medium (lower general secondary education or vocational training); high (pre-university education, high vocational training, university)

### SPN symptoms

At 1-year follow-up, the most frequently reported SPN symptoms were tingling in the fingers/hands (65% experienced some level of symptoms, ranging from “a little bit” to “very much”), tingling in toes/feet (54%), numbness in toes/feet (46%), and numbness in fingers/hands (37%).

Shooting or burning pain was experienced to some degree in the fingers or hands by 19% of patients, and in the toes or feet by 25%. Patients least frequently experienced trouble to distinguish temperature from hot or cold (10%). On average, patients experienced 3.2 symptoms (SD = 1.9) at 1-year follow-up.

The questionnaire at 2-year follow-up was completed by 120 patients that were included in this sample (85%). Of these patients (whom all experienced some level of SPN at 1-year follow-up), 86 (72%) still experienced SPN. The most frequently reported SPN symptoms at 2-year follow-up were the same as the most frequently reported symptoms reported 1 year earlier: tingling in the fingers/hands (58% experienced some level of symptoms, ranging from “a little bit” to “very much”), tingling in toes/feet (56%), numbness in toes/feet (44%), and numbness in fingers/hands (53%). Shooting or burning pain was experienced to some degree in the fingers or hands by 22% of patients, and in the toes or feet by 36%. Patients still least frequently experienced trouble to distinguish temperature from hot or cold (8%) at 2-year follow-up. On average, patients experienced 3.5 symptoms (SD = 2.0) at 2-year follow-up.

### Optimism as a predictor of SPN symptom severity and HRQoL

At 1-year follow-up, optimism was not significantly associated with SPN severity (Table 2). On the contrary, when entered solely into the analyses, optimism was significantly associated with all HRQoL functioning scales and global QoL. These associations were of the smallest magnitude between optimism and role functioning (beta = 0.22, *p* < 0.05). The largest associations existed between optimism and emotional functioning (beta = 0.37, *p* < 0.001). Explained variance in dependent variables by optimism ranged from 4 (role functioning) to 13% (emotional functioning). These significant associations between optimism scores and HRQoL-related dependent variables remained significant when demographic and clinical control variables were added to the analyses for global HRQoL as well as emotional, physical, and cognitive functioning. The same associations became marginally significant for role and social functioning. Importantly, all significant associations between optimism scores and dependent variables became non-significant after controlling for depressive and anxiety symptoms. Most notably, depressive symptoms were consistently and most strongly associated with all HRQoL outcome variables as well as SPN severity (beta’s ranging from 0.23 for SPN severity scores (*p* < 0.05) to beta =  − 0.47 for physical functioning, all other p values < 0.01). Anxiety symptoms were significantly associated with emotional functioning (beta =  − 0.56, *p* < 0.001) and cognitive functioning (beta =  − 0.39, *p* < 0.001). The change in explained variance by adding depressive and anxiety symptoms to the model ranged from 1 (SPN severity) to 36% (emotional functioning).

**Table 2:** Outcomes of multivariable stepwise regression analyses of the association of optimism (LOT-R) with SPN symptom severity and HRQoL among CRC patients with (CI)PN at 1-year follow-up (*n* = 142)

	SPN severity		Global HRQoL		Physical functioning		Role functioning		Emotional functioning		Social functioning		Cognitive functioning	
	Beta	Adj R^2^	Beta	Adj R^2^	Beta	Adj R^2^	Beta	Adj R^2^	Beta	Adj R^2^	Beta	Adj R^2^	Beta	Adj R^2^
Step 1														
LOT-R optimism	.05		.26^*^		.30^**^		.22^*^		.37^**^		.23^*^		.28^**^	
		− .01		.06^a^		.08^a^		.04^a^		.13^a^		.05^a^		.07^a^
Step 2														
LOT-R optimism	.03		.28^*^		.27^*^		.23^x^		.37^**^		.22^x^		.34^**^	
Age	− .21^x^		.11		− .03		.14		.24^*^		− .02		.23^*^	
Sex (female)	− .12		− .06		− .14		− .03		− .08		− .13		− .02	
Education level (high)	− .20^x^		.01		.12		.11		.13		.10		− .09	
Partner (yes)	.04		− .14		− .12		− .11		− .24^*^		− .07		− .01	
Stage (III and IV)	.25^*^		.02		− .06		− .09		.10		− .17		− .02	
		.11^a^		.05		.09		.06		.23^a^		.06		.11
Step 3														
LOT-R optimism	.08		.00		.06		.03		− .06		− .06		− .01	
Age	− .20^x^		.07		− .06		.11		.19*		− .05		.20^*^	
Sex (female)	− .09		− .04		− .15		− .04		.04		− .09		.06	
Education level (high)	− .21^x^		.03		.14		.14		.13^x^		.11		− .09	
Partner (yes)	.04		− .08		− .09		− .17^x^		− .11		− .10		.10	
Stage (III and IV)	.29^**^		− .08		− .15		− .07		.01		− .15		− .10	
Depressive symptoms	.23^x^		− .44^**^		− .47^**^		− .43^**^		− .25^*^		− .35^*^		− .25^*^	
Anxiety symptoms	− .14		− .08		− .08		.05		− .56^**^		− .19		− .39^**^	
		.12		.21^a^		.21^a^		.16^a^		.59^a^		.20^a^		.33^a^

Abbreviations *LOT-R*, Life Orientation Test-Revised; *(S)PN*, (sensory) peripheral neuropathy; *CRC*, colorectal cancer; *HRQoL*, health-related quality of life; *Adj*, adjusted

^*^Significant at *p* < .01

^**^Significant at *p* < .001

^x^Marginally significant at *p* < .05

^a^Change in adjusted R^2^ is significant at *p* < .01. Reported beta’s are standardized coefficients

Further multivariable stepwise regression analyses were performed in the subsample of patients who also completed the questionnaire at 2-year follow-up and still experienced SPN (*n* = 86) to assess the prospective association between optimism (and control variables) at 1-year follow-up and all outcome variables 1 year later. The outcomes revealed that—when entered solely into the analyses—optimism scores at 1-year follow-up were significantly associated with several HRQoL scales at 2-year follow-up (Table 3). These included emotional functioning (beta = 0.42, *p* < 0.001) and cognitive functioning (beta = 0.34, *p* < 0.01), and were marginally significant for global HRQoL (beta = 0.26, *p* < 0.05). The optimism scores were not prospectively associated with the severity of SPN symptoms 2 years after diagnosis, and neither to physical, role, or social functioning scores. The explained variance in significantly associated dependent variables by optimism ranged from 7 (global HRQoL) to 17% (emotional functioning). Similar to the cross-sectional findings, the significant associations between optimism and HRQoL scales were retained when controlling for demographic and clinical variables, but became non-significant after controlling for depressive and anxiety symptom scores. Depressive symptoms at 1-year follow-up were significantly associated with global HRQoL at 2-year follow-up as well as physical, emotional, social, and cognitive functioning (beta’s ranging from − 0.40 for social and cognitive functioning to beta =  − 0.70 for global QoL, all p values < 0.01). Anxiety symptoms were prospectively, marginally significantly associated with social functioning (beta =  − 0.38, *p* < 0.05) and role functioning (beta =  − 0.38, *p* < 0.05). The change in explained variance by adding depressive and anxiety symptoms to the model ranged from 0 (SPN severity) to 37% (emotional functioning).

**Table 3:** Outcomes of multivariate stepwise regression analyses of the prospective association of optimism (LOT-R) at 1-year follow-up with SPN symptom severity and HRQoL at 2-year follow-up among CRC patients with chronic (CI)PN (*n* = 86)

	SPN severity		Global HRQoL		Physical functioning		Role functioning		Emotional functioning		Social functioning		Cognitive functioning	
	Beta	Adj R^2^	Beta	Adj R^2^	Beta	Adj R^2^	Beta	Adj R^2^	Beta	Adj R^2^	Beta	Adj R^2^	Beta	Adj R^2^
Step 1														
LOT-R optimism	− .04		.26^x^		.15		.07		.42^**^		.13		.34^*^	
		− .01		.07^x^		.01		− .01		.17^a^		.00		.10^a^
Step 2														
LOT-R optimism	− .03		.25^x^		.16		.05		.42^**^		.14		.33^*^	
Age	− .15		.05		− .21		.03		.12		.00		.07	
Sex (female)	.03		− .10		− .11		− .03		− .13		− .19		.05	
Education level (high)	− .20		.05		.05		.16		− .06		.06		− .01	
Partner (yes)	.14		− .12		− .02		− .07		− .26^x^		− .19		− .03	
Stage (III and IV)	.11		.03		− .01		− .05		.13		− .02		− .05	
		.00		.02		.01		− .05		.21		.00		.05
Step 3														
LOT-R optimism	.11		− .16		− .14		− .16		.00		− .29^x^		− .08	
Age	− .11		− .06		− .29*		− .08		.07		− .09		− .02	
Sex (female)	.02		− .04		− .07		.04		.01		− .09		.15	
Education level (high)	− .20		.08		− .06		.18		− .03		.08		.01	
Partner (yes)	.12		− .04		.04		− .19		− .18^x^		− .11		.06	
Stage (III and IV)	.16		− .12		− .12		.01		− .05		− .14		− .18	
Depressive symptoms	.22		− .58^**^		− .46^*^		− .54*		− .70^**^		− .40^*^		− .40^*^	
Anxiety symptoms	− .03		− .16		− .07		− .20		− .08		− .38^x^		− .38^x^	
		− .01		.29^a^		.15^a^		.23^a^		.59^a^		.30^a^		.36^a^

Abbreviations *LOT-R*, Life Orientation Test-Revised; *(S)PN*, (sensory) peripheral neuropathy; *CRC*, colorectal cancer; *HRQoL*, health related quality of life; *Adj*, adjusted

^*^Significant at *p* < .05

^**^Significant at *p* < .01

^***^Significant at *p* < .001

^a^Change in adjusted R^2^ is significant at *p* < .05. Reported beta’s are standardized coefficients and outcomes of step 2 of multivariate regression analyses

## Discussion

Using data from a prospective cohort study, we examined if dispositional optimism among CRC patients with (CI)PN was cross-sectionally and prospectively associated with SPN symptom severity and HRQoL one and 2 years after diagnosis. Most frequently reported were tingling and numbness in the fingers/hands and toes/feet, which corresponds to previous findings [3, 23]. Dispositional optimism was indeed related to HRQoL at both time-points, but not to SPN symptom severity. Patients with higher levels of optimism reported better global HRQoL as well as physical, emotional, and cognitive functioning at 1-year follow-up. Furthermore, higher levels of optimism were prospectively related to emotional and cognitive functioning 1 year later. After controlling for depressive and anxiety symptoms, optimism was not associated anymore with any of the outcomes in the study.

To our best knowledge, this is the first study to focus on the role of optimism in the (CI)PN experience. Our findings on the association between optimism and HRQoL are in line with previous studies that reported that more optimistic cancer patients experience higher levels of HRQoL (27–29). It is likely that optimists are able to maintain higher levels of HRQoL in the context of chronic (CI)PN due to their tendency to maintain goal-directed efforts in challenging circumstances, and the consequential use of more adaptive and less nonadaptive coping styles [19]. Optimism was most strongly and consistently over time associated with emotional and cognitive functioning in the context of (CI)PN. Emotional functioning is the aspect of HRQoL that is mostly closely aligned to optimism and the other psychological predictors in our study. In addition, optimism can protect against cognitive impairment via multifactorial, biobehavioral routes. For example, in older adults, these routes may include psychological, biological, and lifestyle processes (e.g., less smoking, lower psychological distress, lower inflammation) [29].

We did expect, however, that optimism would also be related to the self-reported severity of SPN symptoms. Our hypothesis was based more generally on outcomes of previous studies with cancer patients with chronic (CI)PN that reported an association between psychological predictors that are related with optimism, and the onset or severity of persistent neuropathic symptoms [11, 14, 15]. More specifically, we based our findings on the outcomes of a recent meta-analysis that included cancer patients and reported on a significant association between optimism and pain intensity in a range of experimental and clinical settings [18]. In general, all psychological predictors included in our study (optimism as well as depressive and anxiety symptoms) were not (significantly) associated to (CI)PN. The rationale to expect an association between psychological and physiological factors like neuropathic symptoms stems from the neuromatrix theory of pain that recognizes that pain is not purely a direct response to sensory input. It is the consequence of the output of a widely distributed neural brain network that integrates sensory, cognitive-evaluative, motivational-affective, and physiological, homeostatic responses to injury and chronic stress [13]. Our findings might be explained by the fact that we did not specifically focus our study on individuals with painful (CI)PN. As in other studies focusing on colorectal cancer patients treated primarily with oxaliplatin, only a subset of our sample reported painful neuropathic symptoms [23, 30]. The neuromatrix theory of pain may not uphold for peripheral neuropathy symptoms other than pain. Also, first studies indicate that painful (CI)PN differentially impacts HRQoL compared with non-painful (CI)PN [30, 31]. More research on painful (CI)PN is thus necessary, among others to see if psychosocial predictors that include optimism relate differently to (non-)painful (CI)PN onset and severity, and subsequent HRQoL.

It is an important finding that depressive and anxiety symptoms were associated with the study outcomes. These findings corroborate existing studies with cancer patients with chronic (CI)PN. For example, Lee and colleagues showed that pre-treatment anxiety exacerbated the chances to suffer from persistent (CI)PN 8 months after the completion of chemotherapy in women with breast cancer [14]. Another study showed that depressive and anxiety symptoms mediated (CI)PN-related fatigue in a sample of CRC patients [10]. In our findings, the cross-sectional and prospective association between optimism and HRQoL became non-significant after the inclusion of psychological distress in our models. Indeed, previous studies consistently reported that optimism and psychological distress are related in cancer and other health conditions [17, 32]. We propose that the overlap between optimism and psychosocial distress can be explained by the fact that people with psychological distress and low levels of optimism in the context of (CI)PN share the use of non-adaptive coping styles, like avoidance coping, catastrophizing, and a lack of acceptance [19, 33]. These coping styles are key factors in established psychosocial theoretical models that explain how and why people (fail to) adapt to musculoskeletal chronic pain conditions [12, 31]. Theoretical models, such as the Fear Avoidance model and Psychological Inflexibility model of chronic pain, may therefore be used in the design of future studies, to be able study the psychosocial mechanisms underlying chronic (CI)PN and associated impairments in HRQoL in more detail [12, 34].

A first study limitation is the lack of clinician-based assessment of (CI)PN. Patient-reported assessments of (CI)PN should be preferably combined with the use of neurological assessment tools by clinicians, as both provide complimentary but different information [25]. We did use the most commonly implemented patient-reported assessment of (CI)PN which has good psychometric properties, and adhered to recent guidelines that suggest to take caution with the implementation of the EORTC QLQ-CIPN20 item on hearing problems [25]. Furthermore, we had no access to data on type of chemotherapy and cumulative dosage received, which are important predictors of (CI)PN [2]. Furthermore, we cannot guarantee that participants were not receiving any cancer treatment during follow-up measurements. Regular, initial treatment for colon or rectal cancer would have been finished prior to 1-year follow-up. We did not ask during follow-up measurements, however, if participants in the study received a new cancer diagnosis and/or subsequent treatment. Finally, we do not know whether the patients lost to follow-up stopped as they experienced severe PN symptoms (in their hands) or high (clinical) levels of depressive symptoms. If so, this may have skewed our sample and influenced our results. Despite these limitations, our data comes from the first prospective cohort study that examined the association between optimism, (CI)PN, and HRQoL among CRC patients up to 2 years after diagnosis [20].

In conclusion, this longitudinal cohort study showed that dispositional optimism among CRC patients with (CI)PN was cross-sectionally as well as prospectively related to several domains of HRQoL, but not to SPN symptom severity. After controlling for depressive and anxiety symptoms, optimism was not associated anymore with any of the outcomes in the study. Future research may target the examination of differential associations of (non)painful (CI)PN with relevant predictors like optimism, and outcomes. In addition, future research may also shed light on the psychological mechanisms that optimism and psychological distress share, like the use of (non)adaptive coping styles such as avoidance and acceptance. This knowledge may contribute in the long-term to the design of targeted psychosocial interventions to help patients to adapt better to chronic (CI)PN.

## Data Availability

The data that support the findings of this study are available from the corresponding author upon reasonable request.
